# BcTFIIIA Negatively Regulates Turnip Mosaic Virus Infection through Interaction with Viral CP and VPg Proteins in Pak Choi (*Brassica campestris* ssp. *chinensis*)

**DOI:** 10.3390/genes13071209

**Published:** 2022-07-06

**Authors:** Rujia Zhang, Changwei Zhang, Shanwu Lyu, Huiyuan Wu, Mengguo Yuan, Zhiyuan Fang, Fangfang Li, Xilin Hou

**Affiliations:** 1 State Key Laboratory of Crop Genetics & Germplasm Enhancement, Ministry of Agriculture and Rural Affairs, Nanjing 210095, China; 2018204020@njau.edu.cn (R.Z.); changweizh@njau.edu.cn (C.Z.); shanwu.lyu@hotmail.com (S.L.); 2019104075@njau.edu.cn (H.W.); 2020104077@stu.njau.edu.cn (M.Y.); 2 Key Laboratory of Biology and Genetic Improvement of Horticultural Crops (East China), Engineering Research Center of Germplasm Enhancement and Utilization of Horticultural Crops, Nanjing Agricultural University, Nanjing 210095, China; 3 Key Laboratory of Biology and Genetic Improvement of Horticultural Crops, Ministry of Agriculture, Institute of Vegetables and Flowers, Chinese Academy of Agricultural Sciences, Beijing 100081, China; fangzhiyuan@caas.cn; 4 State Key Laboratory for Biology of Plant Diseases and Insect Pests, Institute of Plant Protection, Chinese Academy of Agricultural Sciences, Beijing 100193, China; ffli@ippcaas.cn

**Keywords:** BcTFIIIA, turnip mosaic virus, disease resistance, pak choi

## Abstract

TFIIIA is a zinc-finger transcription factor that is involved in post-transcriptional regulation during development. Here, the *BcTFIIIA* gene was isolated from pak choi. Sequence analysis showed that *BcTFIIIA* encodes 383 amino acids (aa) with an open reading frame (ORF) of 1152 base pairs (bp). We investigated the subcellular location of BcTFIIIA and found the localized protein in the nucleus. *BcTFIIIA* was suppressed when the pak choi was infected by the turnip mosaic virus (TuMV). The *BcTFIIIA* mRNA expression level in a resistant variety was higher than that in a sensitive variety, as determined by qRT-PCR analysis. Yeast two hybrid (Y2H) assay and bimolecular fluorescence complementation (BiFC) suggested that BcTFIIIA interacts with TuMV CP and VPg in vivo, respectively, and in vitro. A virus-induced gene silencing (VIGS) experiment showed that the silencing of *BcTFIIIA* gene expression in pak choi promoted the accumulation of TuMV. These results suggest that BcTFIIIA negatively regulates viral infection through the interaction with TuMV CP and VPg.

## 1. Introduction

Plant viruses are a source of biotic stress that hinders the safe production of agricultural products, affects the yield and quality of agricultural products, and causes significant economic losses [1]. The turnip mosaic virus (TuMV), a member of the Potyvirus genus, has an extremely diverse broad host range of plant species [2]. It is particularly harmful to brassica plants in Asia and has caused severe losses of brassica crops, including oilseed [3] and Chinese cabbage [4]. Several genes were identified to be involved in TuMV resistance, such as *eiF(iso)4e* [5], *NBR1* [6], and *BeClin1* [7]. For viral intercellular mobility and systemic infection in plants, the viral coat protein (CP) is necessary [8,9]. The TuMV CP interacts with AtAP2β. When AtAP2β was overexpressed, TuMV replication was promoted in *Arabidopsis thaliana* [10]. The viral genome-linked protein (VPg) is a multifunctional protein involved in viral genome translation and replication [11]. The TuMV VPg interacts with eiF(iso)4E, and this interaction is necessary for Potyvirus infection [12,13].

One of the largest families of transcription factors in eukaryotes, zinc-finger transcription factors, are involved in the control of biotic and abiotic stress, hormone responses, and plant growth and development [14,15,16]. According to reports, a number of genes play a role in how plants regulate stress. The overexpression of *ZFP36* in rice plants can increase the activity of antioxidant enzymes, and enhance the tolerance of rice plants to water stress and oxidative stress [17]. When SlCZFP1 was overexpressed, Arabidopsis and rice were more resistant to cold stress when *SlCZFP1* was expressed [18]. Phytophthora nicotianae disease resistance requires the expression of *NbCZF1*. Phytophthora nicotianae biomass was considerably higher in NbCZF1-silenced plants compared to potato virus X control plants [19]. OsZFP interacts with southern rice black-streaked dwarf virus (SRBSDV) P8 and is crucial for the infection of the fijivirus and the emergence of symptoms [20].

TFIIIA has several zinc-finger domains and is required in the transcription of 5S rRNA genes. The TFIIIA protein was characterized, and sequence analysis was undertaken in *Xenopus oocytes*, *Saccharomyces cerevisiae*, *Channel catfish oocytes*, *Homo sapiens*, *Bufo americanus*, *Rana pipien*, and Arabidopsis [21,22,23,24,25,26]. Recent studies showed *TFIIIA* to be involved in abiotic stress, such as that due to salt and drought [27], and biotic stress [28]. The overexpression of *TFIIIA*-related transcription factor in the genotype of *Medicago* can increase the salt tolerance of plants [29]. Ribosomal protein L5 and TFIIIA also bind potato spindle tuber viroid (PSTVd) RNA to assist in the synthesis and transport of PSTVd RNA [30].

To date, few studies have been conducted on the functions of the zinc-finger transcription factors in response to abiotic stresses. The functions of the roles of zinc-finger transcription factors during biotic stress are mostly unknown, such as in TuMV infection. According to a previous experiment, BcTFIIIA may interact with CP and VPg by Y2H screen assay. Therefore, the purpose of this study was to evaluate the function of the *BcTFIIIA* gene in cruciferous plants’ resistance to TuMV, and to offer evidence for the possible use of *BcTFIIIA* in enhancing disease resistance. The expression of *BcTFIIIA* under biotic stress and the disease resistance of *BcTFIIIA*-silenced plants were analyzed. In this study, the *BcTFIIIA* gene was cloned from pak choi. TuMV infection inhibited BcTFIIIA, according to the qRT-RCR experiment, and the accumulation of TuMV was promoted in *BcTFIIIA*-silenced pak choi plants. These findings imply that the expression of *BcTFIIIA* reduces viral infection and improves the resistance of plants.

## 2. Materials and Methods

### 2.1. Plant Materials and Growth Conditions

Three pak choi varieties (49CX, resistant variety NHCC001, and sensitive variety NHCC003) were used in this study. When the sprouts developed, the seeds were transplanted onto trays with 32 holes in the substrate (roseate/peat soil = 1:3 (*v*/*v*)) that had been spread out in a plastic Petri dish with moist filter paper. All plants were grown in a climate-controlled room with regulated environmental factors in a climatic room under controlled environmental conditions (photoperiod: 16 h light/8 h dark, temperature: 22 °C/18 °C, and humidity: 60–65%).

*Nicotiana benthamiana* plants were sown in trays with 32 holes with a substrate (roseate/peat soil = 2:3 (*v*/*v*)) and employed for the transient transformation assay. The growth conditions were the same as those for pak choi.

### 2.2. Cloning and Sequence Analysis of BcTFIIIA

The coding sequence of *BcTFIIIA* was retrieved from a database and amplified using certain primers (Table S1) [31]. The amplification product was inserted into a pEASY-Blunt Zero Cloning Vector (TransGen Biotech, Beijing, China) for sequencing. Sequence alignments were carried out using BioXMv2.6 software to screen the right clone.

The physicochemical characteristics of BcTFIIIA were anticipated using the Expasy website (https://web.expasy.org/protparam/, accessed on 10 May 2021). The molecular mass of BcTFIIIA was calculated on a website (https://www.novopro.cn/tools/protein_mw.html, accessed on 22 May 2021). The distribution of conserved motifs was analyzed on the MEME website (https://meme-suite.org/meme/tools/meme, accessed on 22 May 2021).

### 2.3. Accession Numbers

The NCBI website has the sequence information from this paper under accession numbers: BnTFIIIA (XM_013836160), BoTFIIIA (XM_013760871), RsTFIIIA (XM_018590742), EsTFIIIA (XM_006390649), CsTFIIIA (XM_010429758), CrTFIIIA (XM_006301733), AtTFIIIA (AT1G72050), MtTFIIIA (LOC120580532), SlTFIIIA (NC_015447), HaTFIIIA (LOC110886130), ZmTFIIIA (LOC100383605), and BcTFIIIA.

### 2.4. Subcellular Localization of BcTFIIIA

The *BcTFIIIA* fragment with the gateway joint was amplified from the pEASYBlunt vector using primers gatewayBcTFIIIA-F and gatewayBcTFIIIA-R, and then introduced into entrance vector pDONR221. This was performed to confirm BcTFIIIA’s subcellular localization. Following *Mlu*I enzyme digestion, the gateway cloning technique was used to subclone BcTFIIIA into the pEarlyGate104 vector (Invitrogen, Carlsbad, CA, USA). *Agrobacterium tumefaciens* (GV3101 strain) was transformed with recombinant plasmid 35S: yellow fluorescent protein (YFP)-BcTFIIIA and empty vector plasmid 35S: YFP. Then, each was injected into the foliar epidermis of the *N. benthamiana* plant. At 48–72 h following agrobacterium injection, fluorescence was seen using a confocal laser scanning microscope (Zeiss, LSM 780, Jena, Germany) outfitted with a 20× water-corrected objective in multitrack mode. YFP was stimulated at 514 nm, and collected between 565 and 585 nm; the same conditions were used for the confocal images reported in the same graph panel.

### 2.5. Yeast Two-Hybrid Assays

In order to create AD–BcTFIIIA fusions, the coding sequences of BcTFIIIA that were amplified by primers called BcTFIIIA-EcoRI-AD-F and BcTFIIIA-BamHI-AD-R (Table S1) and cloned into a pGADT7 vector using the ClonExpress^®^ II One Step Cloning Kit (Vazyme, Nanjing, China). The fusion construct (AD–BcTFIIIA) and 11 viral proteins fused with pGBKT7 (BD-P1, BD-HcPro, BD-P3, BD-PIPO, BD-6K1, BD-CI, BD-6K2, BD-VPg, BD-NIa, BD-NIb, BD-CP), plasmid activation domain (AD), and binding domain (BD) as a negative control, and plasmids pGBKT7-P53 and pGADT7-T as a positive control were transformed into Golden Yeast (Clontech, Dalian, China) cells through the lithium acetate-mediated method. To check for positive interactions, three days after the transformation, the transformed yeast strains were cultured on an SD/-Trp-Leu (Clontech, China) medium and SD/-Trp-Leu-His-Ade (Clontech, China) media at 10-fold serial dilutions. The empty vector (AD) and 11 viral proteins fused with pGBKT7 (BD-P1, BD-HcPro, BD-P3, BD-PIPO, BD-6K1, BD-CI, BD-6K2, BD-VPg, BD-NIa, BD-NIb, BD-CP) were transformed into Golden Yeast (Clontech, China) cells to test the autoactivation of viral proteins.

### 2.6. Bimolecular Fluorescence Complementation

After *Mlu*I enzyme digestion, the gateway cloning technique (Invitrogen, Carlsbad, CA, USA) was used to subclone the *BcTFIIIA* coding sequence into the pEarleygate202 vector that included the N-terminal half of YFP (YN). The coding sequences of CP and VPg were subcloned into the pEarleygate202 vector that contained the C-terminal half of YFP (YC) after *Mlu*I enzyme digestion by the gateway cloning system (Invitrogen, Carlsbad, CA, USA). Plasmid YN-BcTFIIIA was transformed into Agrobacterium (GV3101 strain) and mixed with YC-CP and VPg, which were infiltrated into *N. benthamiana*. Fluorescence was observed with a confocal laser scanning microscope (Zeiss, LSM 780, Jena, Germany) at 48–72 h after Agrobacterium infiltration.

### 2.7. Virus-Induced BcTFIIIA Gene Silencing in Pak Choi Plants

VIGS was performed according to a previous report with slight modifications [32]. The 40 bp *BcTFIIIA*-specific DNA fragment and its antisense sequence (5′–TTGAAAAAGAACATCAAGAGACATCTACGGACTCATGAAGCTTCATGAGTCCGTAGATGTCT CTTGATGTTCTTTTTCAA–3′) were synthesized and subcloned into the *SnaB*I site of the PTY vector by the GeneScript Company (Nanjing, China). Gold particles were covered with PTY-S and PTY-*BcTFIIIA* plasmids, and transformed into the two-week-old seedlings of pak choi by particle bombardment. For every gun, four to six plants were bombarded, and three copies were produced. The newborn leaves began to exhibit clear PTY viral symptoms two weeks later. To determine if the gene was silenced, samples of freshly sprouted leaves were collected, and the qRT-PCR experiment was performed.

### 2.8. TuMV Inoculation Procedure

The procedure of TuMV infiltration was according to the previous study [33]. TuMV-GFP was infiltrated when *N. benthamiana* formed 4–5 true leaves via Agrobacterium infection [7]. After two weeks, the symptoms of the plants could be used to determine whether the infection had been successful. The affected leaves were then picked off and ground into a paste. The paste was filtrated through gauze, and the sap was infiltrated into 49CX. This experiment was repeat at least three times, and 10 plants were used once.

### 2.9. RNA Isolation and Quantitative Real-Time PCR Analysis

TIANGEN’s plant RNA extraction kit was used to extract the total RNA from leaves according to the protocol, and the complementary DNA (cDNA) was synthesized with the Evo M-MLV RT Kit with gDNA Clean for qPCR (Accurate Biology, Changsha, China) according to the instructions. Three biological and technical replicates were used in quantification real-time PCR experiments. The system refers to the Hieff^®^ qPCR SYBR Green Master Mix protocol (High Rox Plus) (YEASEN, Shanghai, China).

BcTFIIIA-specific primers qBcTFIIIA-F and qBcTFIIIA-R (Table S1) were designed using the website (https://www.ncbi.nlm.nih.gov/tools/primer-blast/index.cgi?LINK_LOC=BlastHome, accessed on 10 October 2020), and BcActin and BcPP2A were selected as the internal control genes [34]. According to the previously published procedure, the relative expression levels of the chosen transcripts were normalized to either the BcActin or BcPP2A gene and computed using the 2^−∆∆CT^ method [35]. Viral quantification was calculated using the specific primers of TuMV-CP (Table S1) to conduct the real-time PCR experiment.

### 2.10. Data Analysis

Data are presented in this manuscript following normal distribution (N(μ, σ^2^)), and statistical significance was analyzed using Student’s *t*-test. Significance values with *p* < 0.01 are denoted as **.

## 3. Results

### 3.1. Cloning and Sequence Analysis of BcTFIIIA

The BcTFIIIA gene from pak choi was successfully cloned by using PCR. It had a complete 1149 bp CDS. In order to find the right clone, the PCR result was cloned into a pEASY-Blunt Zero Cloning Vector (TransGen Biotech, China). The polypeptide of 383 amino acids were encoded by the mRNA of BcTFIIIA with a predicted molecular mass of 43.89 kDa, and a theoretical pI of 8.25 was computed. BcTFIIIA was identified as an unstable and hydrophilic protein with the computation of the instability index (II) and the grand average of hydropathicity (GRAVY), which were 56.69 and −1.021, respectively.

**Figure 1:**
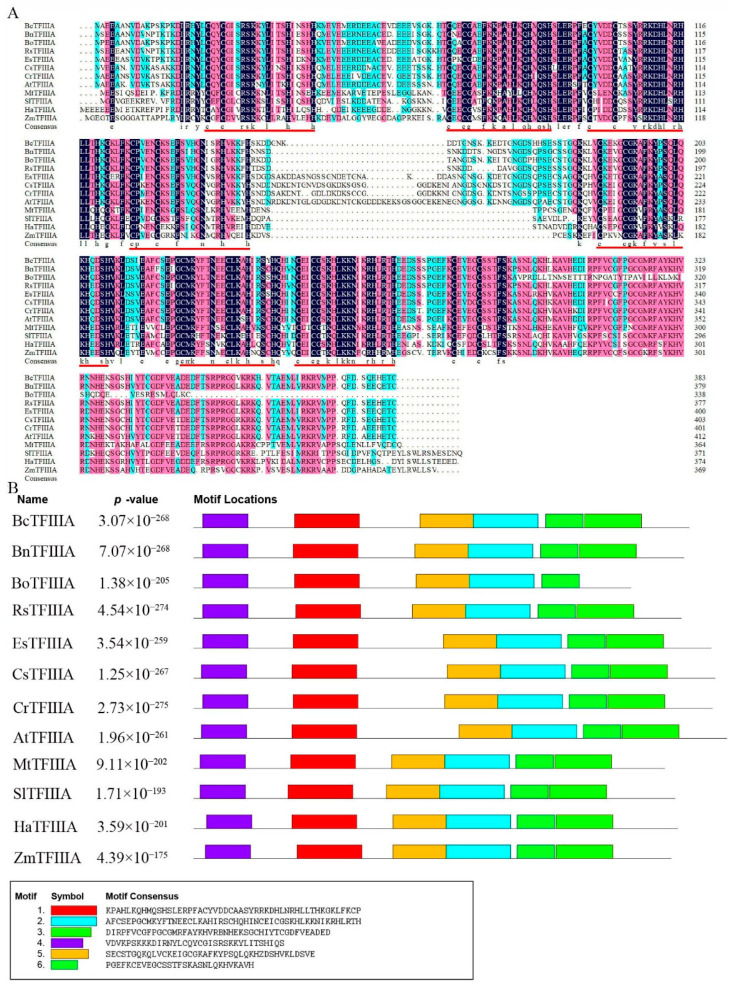
Multiple sequence alignment and motif composition of TFIIIA proteins. (**A**). Sequence alignment of TFIIIA proteins in *Brassica napus*, *Brassica oleracea*, *R. sativus*, *E. salsugineum*, *C. sativa*, *C. rubella*, Arabidopsis, *M. truncatula*, *S. lycopersicum*, *H. annuus*, *Z. mays*, and *B. campestris*. C2H2 zinc finger domains are shown above the red lines. (**B**). Distribution of conserved motifs of TFIIIA. At the bottom, the six motif sequences are displayed; the *p*-value represents the significance of each motif.

Multiple sequence alignment of the TFIIIA from *Brassica napus*, *Brassica oleracea*, *Raphanus sativus*, *Eutrema salsugineum*, *Camelina sativa*, *Capsella rubella*, Arabidopsis, *Medicago truncatula*, *Solanum lycopersicum*, *Helianthus annuus*, *Zea mays*, and *B. campestris* showed that 7 highly conserved C2H2 zinc-finger domains were contained by the BcTFIIIA protein (Figure 1A). BcTFIIIA is a typical zinc-finger protein. More specifically, motifs 1 and 4 located in the N-terminal region, and motifs 2, 5, and 6 located in the C-terminal region are highly conserved in all TFIIIA proteins (Figure 1B).

### 3.2. Phylogenetic Analysis of TFIIIA Proteins

**Figure 2:**
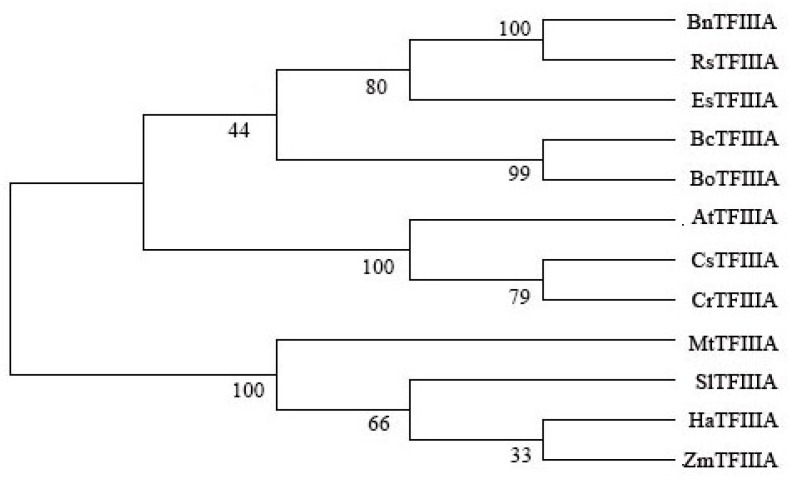
Phylogenetic tree analysis of TFIIIA proteins. The phylogenetic relationship of TFIIIA was constructed using MEGA5.2 software; neighbor-joining was used to construct the phylogenetic tree, and the bootstrap value was set to 1000.

An unrooted neighbor-joining (NJ) phylogenetic tree was built to examine the phylogenetic relationships of TFIIIA proteins using the full-length protein sequences from 12 different species, consisting of 1 monocot, *Zea mays*, and 11 dicots, *Brassica napus*, *Brassica oleracea*, *R. sativus*, *E. salsugineum*, *C. sativa*, *C. rubella*, Arabidopsis, *M. truncatula*, *S. lycopersicum*, *H. annuus*, and *B. campestris*. The TFIIIA proteins were split into two larger groups, and it is possible that the *TFIIIA *genes in pak choi and oilseed both descended from a single ancestral gene. MtTFIIIA forms a separate branch, which indicates that it is different from other species in evolution. BcTFIIIA most strongly resembles BoTFIIIA (Figure 2).

### 3.3. Expression Pattern Analysis of the BcTFIIIA Gene

**Figure 3:**
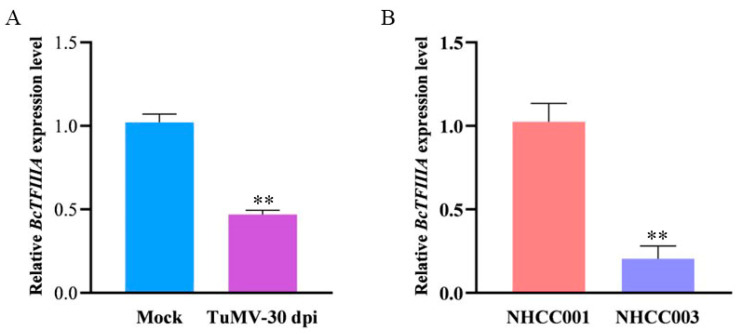
The expression levels of BcTFIIIA in various plants. (**A**) Analysis of *BcTFIIIA* mRNA levels by qRT-PCR in mock (buffer-infected) and TuMV-30 dpi (TuMV-infected 30 dpi) in 49CX. (**B**) Analysis of *BcTFIIIA* mRNA levels in resistant variety NHCC001 and sensitive variety NHCC003 by qRT-PCR. Three plants were used in this experiment. Significant differences were determined by Student’s *t*-test, ** *p* < 0.01.

To detect the effect of TuMV infection on the expression of *BcTFIIIA*, the mRNA levels of *BcTFIIIA* in mock and TuMV-infected 49CX were analyzed. The result demonstrates that, at 30 days postinoculation (dpi), the infected plants had a decreased level of *BcTFIIIA* gene expression (Figure 3A).

According to a previous study, after TuMV inoculation, NHCC003 has obvious symptoms, whereas NHCC001 does not [33]. To verify the role of the BcTFIIIA gene in TuMV resistance, we analyzed the mRNA levels of *BcTFIIIA* in resistant variety NHCC001 and sensitive variety NHCC003 by qRT-PCR. The result shows that *BcTFIIIA* was expressed at higher levels in resistant variety NHCC001 compared to sensitive variety NHCC003 (Figure 3B). These results show that *BcTFIIIA* may suppress TuMV infection in pak choi.

### 3.4. Subcellular Localization of BcTFIIIA Protein

**Figure 4:**
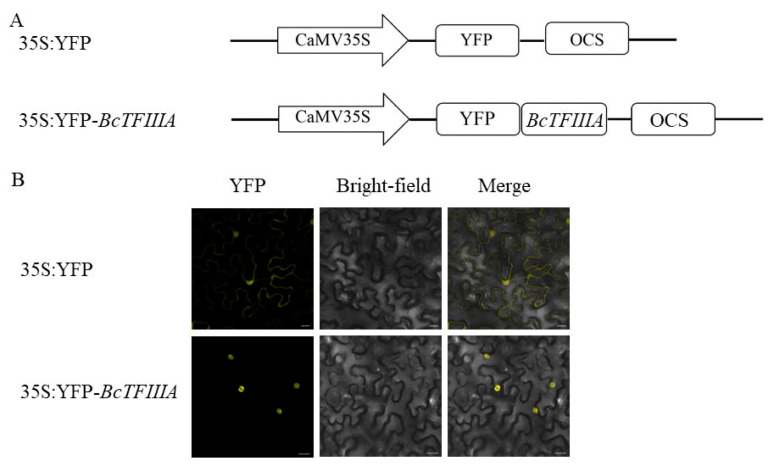
Subcellular localization of BcTFIIIA. (**A**) The construct of 35S:yellow fluorescent protein (YFP) and 35S:YFP–BcTFIIIA fusion protein. (**B**). (left to right) Fluorescence, bright-field, and merged fluorescence images of 35S:YFP and 35S:YFP–BcTFIIIA fusion protein. Bars, 20 µm. Images were collected at 72 hpi after Agrobacterium cultures containing YFP or YFP–BcTFIIIA had been injected onto *N. benthamiana* leaves.

Online analysis in silico (https://wolfpsort.hgc.jp/, accessed on 10 October 2020) indicated that BcTFIIIA might be located in the nucleus. To demonstrate this, we fused BcTFIIIA with 35S:YFP to create a construct (35S: YFP-BcTFIIIA), with vector 35S:YFP serving as a control (Figure 4A). Using Agrobacterium -mediated transformation in tobacco epidermal cells, these two constructs were momentarily expressed. When the control vector was employed, we noticed that the YFP signal was uniformly distributed throughout the nucleus and cytoplasm of epidermal cells. However, only cells producing fusion protein YFP–BcTFIIIA had the fluorescence signal visible in their nucleus (Figure 4B), which indicated that BcTFIIIA targeted to the nucleus. The presence of full-length recombinant proteins was confirmed by Western blotting (WB) with a GFP antibody (@GFP) (Figure S1), which revealed the predicted and precise band corresponding to the YFP–BcTFIIIA and YFP proteins.

### 3.5. BcTFIIIA Interacts with CP and VPg

**Figure 5:**
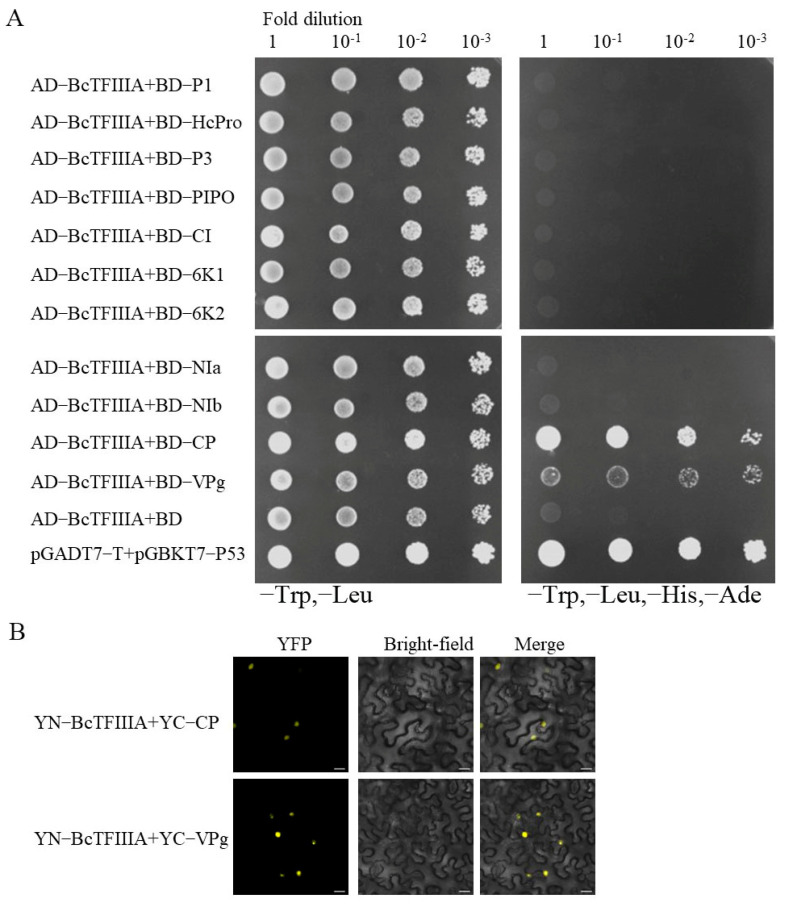
BcTFIIIA interacts with CP and Vpg. (**A**) Y2H assay analysis of potential viral protein interactions with BcTFIIIA. P1, HC−Pro, P3, P3N−PIPO, 6K1, CI, 6K2, VPg, NIa, NIb, or CP (as BD fusion) and BcTFIIIA (as AD fusion) were cotransformed into Y2H Gold yeast cells. Three days After after transformation, the cotransformed proteins were plated onto selective synthetic dextrose (without Trp, Leu, or without Trp, Leu, His, Ade). (**B**) In BiFC assays between BcTFIIIA and CP, VPg in *N. benthamiana* leaves, confocal imaging was observed at 72 h postinoculation (hpi). Viral proteins were fused to the C-terminal segments of yellow fluorescent protein (YFP) (YC), and BcTFIIIA was inserted to the YN. Bars, 20 µm.

We investigated potential interactions between BcTFIIIA and the 11 TuMV proteins using the yeast two-hybrid experiment. The empty vector (AD) and 11 viral proteins fused with pGBKT7 (BD-P1, BD-HcPro, BD-P3, BD-PIPO, BD-6K1, BD-CI, BD-6K2, BD-VPg, BD-NIa, BD-NIb, BD-CP) were transformed into Golden Yeast (Clontech, China) cells to test the autoactivation of viral proteins. The yeast did not grow normally on selective synthetic dextrose (without or Trp, Leu, His, and Ade), which indicates that there was no autoactivation of viral proteins (Figure S2). The Y2H yeast cells coexpressing the TuMV bait proteins with the prey BcTFIIIA grew vigorously on the double-dropout medium deficient in leucine and tryptophan. Each of the 11 viral genes was cloned into the bait vector BD, and BcTFIIIA was cloned into the prey vector AD. In order to look for beneficial interactions three days after transformation, they were serially diluted 10 times and plated on selective synthetic dextrose (without Trp, Leu, or Trp, Leu, His, and Ade). These results show that BcTFIIIA interacts with CP and VPg of the TuMV proteins (Figure 5A). In addition, the interactions between BcTFIIIA and CP, VPg proteins were verified by BiFC. The yellow fluorescence was found in nucleus in cotransformed *N. benthamiana* cells expressing BcTFIIIA-nYFP and CP, VPg-cYFP indicated that BcTFIIIA interacts with CP and VPg (Figure 5B). In conclusion, BcTFIIIA interacted with CP and VPg in the nucleus. VPg is also localized in the nucleus, and BcTFIIIA is located in the nucleus. The nucleus is the site where the virus replicates, and VPg is involved in viral replication, so we speculate that *BcTFIIIA* involves in TuMV replication. The localization of CP is not clear, BcTFIIIA interacts with CP protein in the nucleus may due to BcTFIIIA being localized in nucleus. CP participates in viral movement, which indicates that BcTFIIIA may participate in viral cell-to-cell movement and long-distance movement through these interactions. The nucleus is the site where the virus replicates.

### 3.6. Silencing of BcTFIIIA Facilitated TuMV Infection

**Figure 6:**
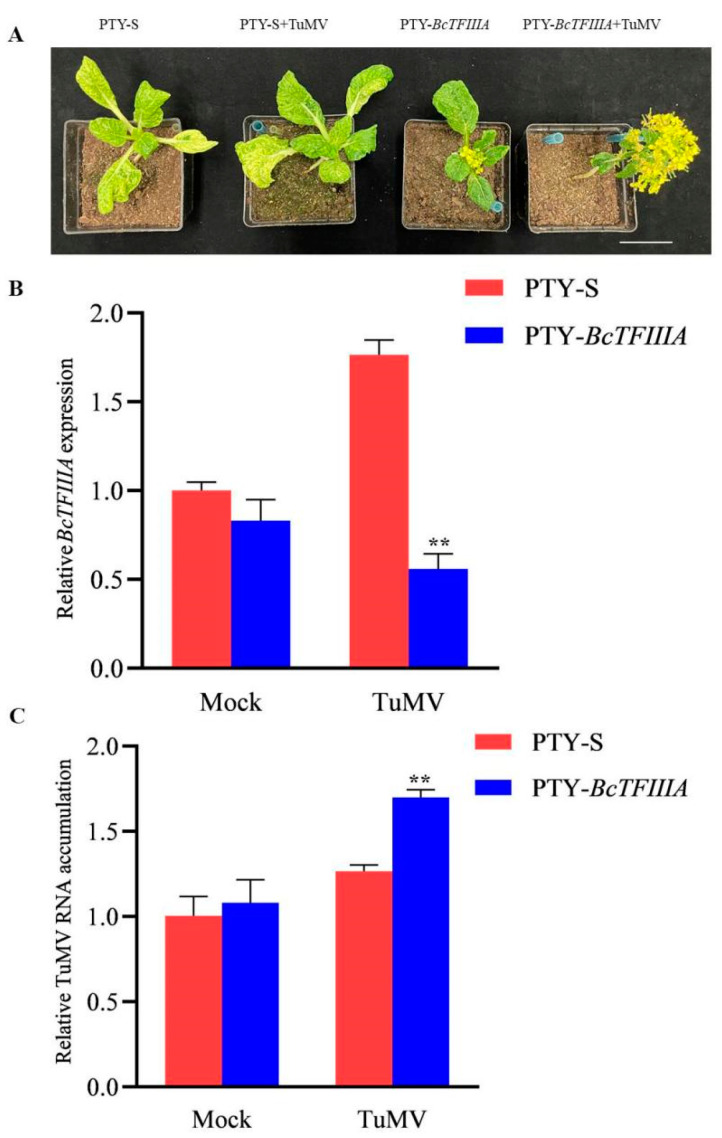
Silencing of *BcTFIIIA* promotes TuMV infection in 49CX. (**A**). Plant images were taken at 20 dpi. The 49CX was preinjected with PTY-S (control), PTY-*BcTFIIIA* (to silence the *BcTFIIIA*) for 10 days, and then injected with TuMV-GFP for 20 days. Bar scale, 5 cm. (**B**). The level of *BcTFIIIA* expression in these plants. RNA was isolated from systemically infected leaves at 20 dpi. (**C**). TuMV RNA accumulation in these plants. Three plants and three experiments were analyzed in this study. Significant differences were determined by Student’s *t*-test, ** *p* < 0.01.

The different transcriptional levels of *BcTFIIIA* between NHCC001 and NHCC003 suggested that *BcTFIIIA* is a negative regulator in promoting TuMV infection. To verify this, the VIGS technique due to the turnip yellow mosaic virus (TYMV) was used to inhibit the expression of *BcTFIIIA*. The 49CX plants were inoculated with PTY-*BcTFIIIA* (to silence *BcTFIIIA*) or PTY-S (as a control) and after 10 days infiltrated with TuMV-GFP in the leaves upper the leaves infiltrated with PTY. The *BcTFIIIA*-silenced plants were more sensitive to TuMV, and the flowering time was advanced as compared to the control plants (Figure 6A). We used qRT-PCR analysis to determine whether *BcTFIIIA* was silenced in plants preinoculated with PTY-*BcTFIIIA*, and we discovered that the expression level of *BcTFIIIA* decreased in these plants (Figure 6B). Compared to the control, higher levels of TuMV RNA accumulation were discovered in the *BcTFIIIA*-silenced plants (Figure 6C). This result agrees with the phenotype. Overall, these results suggest that *BcTFIIIA* participates in anti-TuMV defense, and *BcTFIIIA* negatively regulates TuMV infection.

## 4. Discussion

Cultivating disease-resistant varieties and improving disease resistance have always been the main goals of breeding in crops. TuMV, one of the most harmful vegetable viruses in the world, belongs to the Potyvirus genus [36]. The recently identified zinc-finger transcription factors related to plant disease responses include a part of AP2/ERF family proteins that have the zinc-finger domain [37] and WRKY zinc-finger transcription factor families [38,39,40]. Gene families involved in TuMV resistance, including the TIR-NBS-LRR gene family [41] and the Fasciclin-like arabinogalactan (FLA) gene family [42], have also been identified and researched. 

According to previous studies, abiotic stress responses and plant development are mediated by zinc-finger transcription factors of the TFIIIA type [14,15,29,43]. Nevertheless, the response of TFIIIA-type zinc-finger transcription factors to biotic stress has only been the subject of a small number of studies. Arabidopsis *L5* and *TFIIIA* bind *potato spindle tuber viroid* (PSTVd) RNA in vitro, and participate in the synthesis and delivery of PSTVd RNA in vivo [30]. This indicates that *TFIIIA* influences viral infection. However, the molecular mechanism of the *TFIIIA* gene’s antiviral effect in plants is not clear.

TFIIIA has two variants, containing seven or nine zinc fingers. A previous study showed that the viral interaction with TFIIIA contained seven zinc fingers to influence the infection of the virus [28]. In this study, we discovered a *TFIIIA* gene in pak choi that had seven zinc fingers linked to TuMV resistance. A resistant variety, NHCC001, had a higher level of *BcTFIIIA* gene expression than that of a susceptible variety, NHCC003. Further, the expression level was reduced in TuMV-GFP infected plants compared to normal plants (Figure 3). We speculated that a high transcript level of *BcTFIIIA* in the plants can improve disease resistance. In order to verify this conjecture, we silenced the expression of *BcTFIIIA* in pak choi. The upper newly emerging leaves in *BcTFIIIA* silenced plants contained larger quantities of TuMV genomic RNA. (Figure 6). Conclusively, these findings demonstrate that *BcTFIIIA* contributes to TuMV resistance in pak choi. The expression of *BcTFIIIA* suppressed TuMV infection. However, how *BcTFIIIA* suppressed TuMV infection needs further study. A previous study showed that L5 and TFIIIA bind PSTVd (+) RNA to influence the synthesis and transportation of PSTVd [30], so we suppose that *BcTFIIIA* can interact with *BcL5* and thus influence TuMV infection.

For a new round of infection, a potyvirus must travel from the replication site to the cell’s plasmodesmata, pass through the plasmodesmata, and infiltrate nearby cells [44]. Viral infection, viral amplification, and cap-independent translation are all crucially influenced by VPg [45]. Studies have revealed that VPg functions similarly to a cap by introducing ribosomes and translation initiation components to viral mRNA to facilitate effective translation [46]. CP is involved in viral long-distance movement and cell-to-cell movement [47]. AtDRP2 interacts with TuMV proteins VPg and CP; the TuMV RNA level was reduced in *atdrp2* mutants and increased in AtDRP2-overexpressing plants [48]. Y2H and BiFC assays showed the interaction between BcTFIIIA and CP, VPg (Figure 5). Here, we assume that BcTFIIIA, CP, and VPg interact because of both Y2H and BiFC validation. Since VPg and CP are involved in viral replication and movement [8,49], this indicates that *BcTFIIIA* may participate in TuMV disease resistance and influence viral replication, cell-to-cell movement, and long-distance movement through these interactions. The nucleus is the site where the virus replicates [50]. BcTFIIIA is located in the nucleus (Figure 4), so we speculate that *BcTFIIIA* is involved in TuMV replication. This is a hypothesis and still requires experimental verification.

## 5. Conclusions

In summary, we cloned the *BcTFIIIA* gene from pak choi and identified its response to the TuMV. BcTFIIIA is localized in the nucleus, and the expression of the BcTFIIIA gene decreased after the infection of TuMV with pak choi. Meanwhile, silencing the expression of BcTFIIIA gene promoted the infection of TuMV. BcTFIIIA interacted with CP and VPg. Our study provides new insights into how transcription factor BcTFIIIA from pak choi regulates the response to TuMV infection.

## Data Availability

Not applicable.

## Supplementary Materials

The following supporting information can be downloaded at: https://www.mdpi.com/article/10.3390/genes13071209/s1, Figure S1: Western blot (WB) analysis of total protein. Figure S2: autoactivation test by viral proteins. Table S1: primers used in this study.
