# Supplementary material for: BcTFIIIA Negatively Regulates Turnip Mosaic Virus Infection through Interaction with Viral CP and VPg Proteins in Pak Choi (Brassica campestris ssp. chinensis)
Source: Genes (Basel). 2022 Jul 6;13(7):1209. doi: 10.3390/genes13071209 (PMC9317785; doi:10.3390/genes13071209)
Supplement: Supplementary file 1 [file genes-13-01209-s001.zip › genes-1785059-supplementary.pdf]

Table S1. Primers used in this study

Primer	Sequence (5'-3')	Note
BcTFIIIA-F	ATGGCGGAAGAAGCAGCTAACG	CDS Cloning
BcTFIIIA-R	GCAAGTCTCGTGTTCTTGTGAATC	
gateway BcTFIIIA-F	ggggacaactttgtacaaaaaagttggcATGGCGGAAGAAGCAGCTAACG	For LR reaction
gateway BcTFIIIA-R	ggggacaactttgtacaagaaagttgggcaGCAAGTCTCGTGTTCTTGTGAATC	
BcTFIIIA-EcoRI-AD-F	gccatggaggccagtgaattcATGGCGGAAGAAGCAGCTAACG	For yeast two hybrid
BcTFIIIA-BamHI-AD-R	cagctcgagctcgatggatccccGCAAGTCTCGTGTTCTTGTGAATC	
qBcACTIN-F	GTTGCTATCCAGGCTGTTCT	RT-qPCR
qBcACTIN-R	AGCGTGAGGAAGAGCATAAC	
qBcTFIIIA-F	GAAGCTTGCGAGGTTGATGA	RT-qPCR
qBcTFIIIA-R	GCTCTGCATATGCTGCTTCA	
qBcPP2A-F	AGGCTACACGTTCGGACAAG	RT-qPCR
qBcPP2A-R	TGGGGCACTAAACACAGTCA	
qTuMV-CP-F	TGGCTGATTACGAACTGACG	RT-qPCR
qTuMV-CP-R	CTGCCTAAATGTGGGTTTGG	

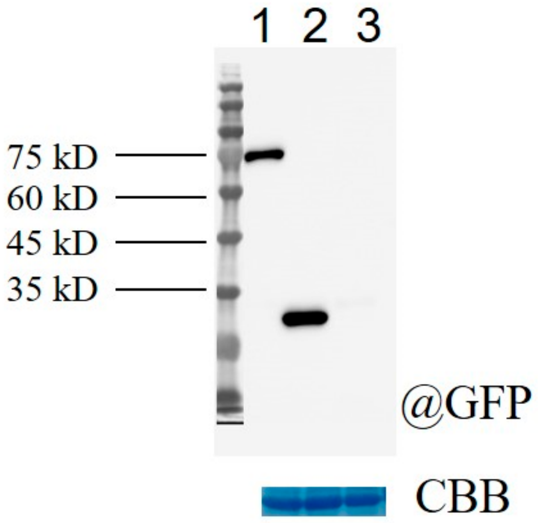

Figure S1. Western Blot (WB) analysis of total protein. Samples from infiltrated leaves were taken and used to conduct an experiment on subcellular localization at 72 hours post infiltration (hpi), antibody against GFP (@GFP) was applied. Coomassie brilliant blue-stained Rubisco large subunit was used as a loading control. 1, 2, 3 means 35S: YFP-BcTFIIIA, 35S: YFP and wild type, respectively.

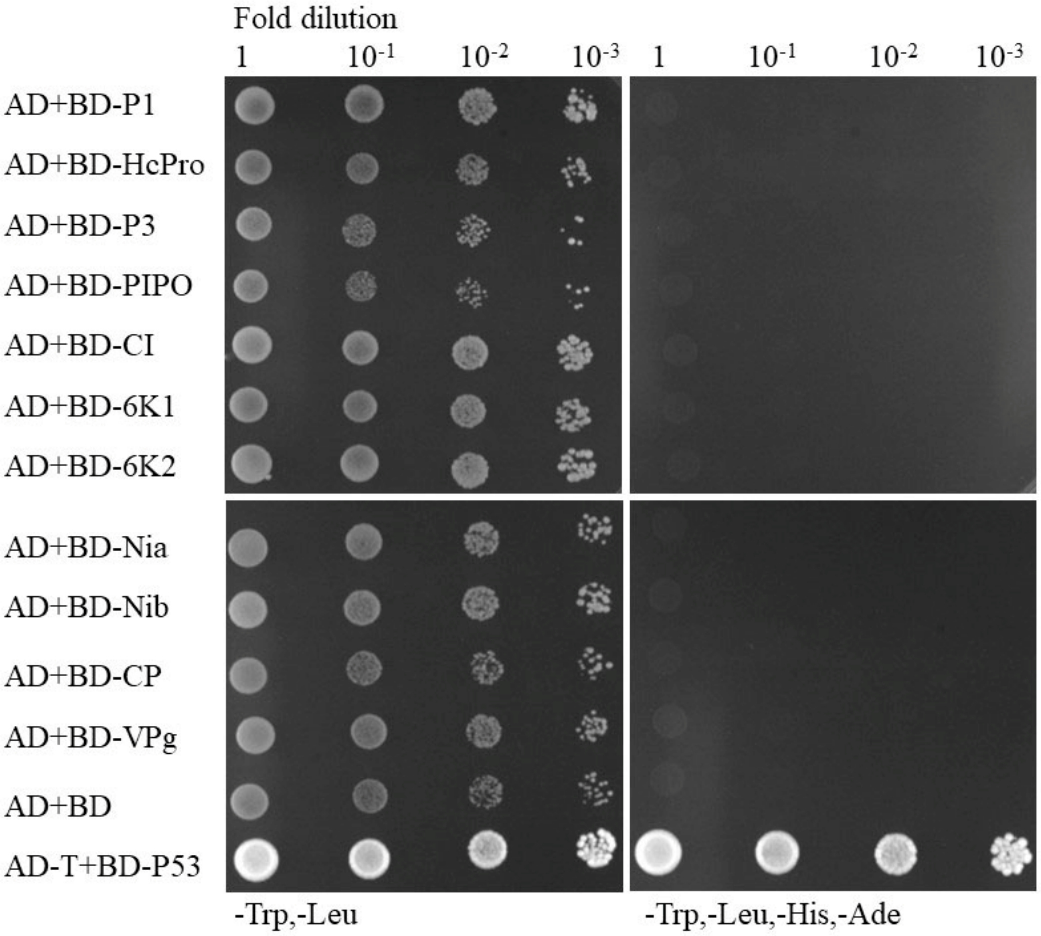

Figure S2. Autoactivation test by viral proteins. The viral proteins were subcloned in the BD (bait vector) and co-transformed in Y2H Gold yeast cells with AD empty vector. After 3 days transformation, the co-transformed proteins were dilluted and plated on selective synthetic dextrose (without Trp, Leu, or without Trp, Leu, His, Ade). We observed whether the yeast is growing normally after 3 days to determine whether the viral proteins is self-activated.
